# 

**Published:** 1857-11

**Authors:** 


### LIST OF COLLABORATORS.

S. G. Armor, M.D., Professor of Pathology and Clinical Medicine, Missouri Med. College.

John L. Atlee, M.D., President Pennsylvania State Medical Society.

Walter F. Atlee, M.D., Philadelphia.

John Bell, M.D., Philadelphia, formerly Professor of Medicine, Medical College of Ohio.

T. S. Bell, M.D., Louisville, Ky.

S. M. Bemiss, M.D., Louisville, Ky.

L. P. Blackburn, M.D., Natchez, Miss.

G. C. Blackman, M.D., Professor of Surgery, Medical College of Ohio.

W. S. Bowen, M.D., New York.

J. L. Bobbs, M.D., formerly Professor of Anatomy, Indianapolis.

W. M. Boling, M.D., Montgomery, Ala., formerly Professor of Midwifery, Transylvania University, Lexington, Ky.

N. Bozeman, M.D., Montgomery, Ala.

J. T. Bradford, M.D., Augusta, Ky.

John H. Brinton, M.D., Lecturer on Operative Surgery, Philadelphia.

W. S. Chipley, M.D., Professor of Medicine, Transylvania University.

A. Clapp, M.D., New Albany, Ia.

D. B. Cliffe, M.D., Franklin, Tenn.

J. Da Costa, M.D., Lecturer on the Practice of Medicine, at the Philad. Med. Association.

M. Dupierris, M.D., Havana, Cuba.

W. F. Edgar, M.D., United States Army.

S. C. Farrar, M.D., Jackson, Miss.

C. S. Fenner, M.D., Memphis, Tenn.

Austin Flint, M.D., Professor of Clinical Medicine and Pathology, University of Buffalo.

Charles Fricke, M.D., Baltimore.

W. Y. Gadbury, M.D., Benton, Miss.

O. C. Gibbs, M.D., Chautauque County, New York.

Dr. J. P. Hall, Glasgow, Ky.

John Hardin, M.D., Professor of Obstetrics, Kentucky School of Medicine, Louisville.

EDWARD HARTSHORNE, M.D., One of the Surgeons to Wills Hospital for Diseases of the Eye, Philadelphia.

E. B. HASKINS, M.D., Clarksville, Tenn., late President of the Tennessee State Medical Society.

C. A. HENTZ, M.D., Marianna, Florida.

ADDINELL HEWSON, M.D., One of the Surgeons to Wills Hospital for Diseases of the Eye, Philadelphia.

SAMUEL L. HOLLINGSWORTH, M.D., late Editor of the Medical Examiner, Philadelphia.

WILLIAM A. HAMMOND, M.D., United States Army.

WILSON JEWELL, M.D., Philadelphia, President of the Quarantine and Sanitary Convention.

G. A. KUNKLER, M.D., Madison, Ia.

WM. V. KEATING, M.D., Lecturer on Obstetrics and the Diseases of Women, in the Philadelphia Medical Association.

BEN. LOGAN, M.D., Shreveport, La.

A. LOPEZ, M.D., Mobile, Ala., formerly President of the Alabama State Medical Society.

GEORGE R. MOREHOUSE, M.D., Philadelphia.

H. WEIR MITCHELL, M.D., Lecturer on Physiology at the Philad. Med. Association.

B. I. RAPHAEL, M.D., New York.

J. C. REEVE, M.D., Dayton, Ohio.

L. C. RIVES, M.D., formerly Professor of Midwifery, Medical College of Ohio.

J. LAWRENCE SMITH, M.D., Professor of Chemistry, University of Louisville.

W. C. SNEED, M.D., Frankfort, Ky., President Kentucky State Medical Society.

C. H. SPILMAN, M.D., Harrodsburg, Ky., late President of Kentucky State Medical Society.

GEO. SUTTON, M.D., Aurora, Ia.

W. L. SUTTON, M.D., Georgetown, Ky., formerly President Kentucky State Medical Society.

HORATIO R. STORER, M.D., formerly one of the Physicians to the Boston Lying-in Hospital, Boston, Mass.

S. THOMPSON, M.D., Albion, Ill.

E. WILLIAMS, M.D., Cincinnati, Ohio.

### Bi-Monthly Bibliographical Record.

### AMERICAN.

General Therapeutics and Materia Medica; adapted as a Medical Text-book, with Indexes of Remedies, and of Diseases and their Remedies. By Robley Dunglison, M.D., LL.D., &c. Sixth Edition, revised and improved; in two vols. 8vo. Philadelphia: Blanchard & Lea, 1857. (Received from the Publishers.)

A Collection of Remarkable Cases in Surgery. By Paul F. Eve, M.D., Professor of Surgery, University of Nashville. Philadelphia: J. B. Lippincott & Co. 1857. 8vo. (Received from the Publishers.)

The Medical Student's Vade Mecum; a Compendium of Anatomy, Physiology, Materia Medica, Practice of Medicine, Surgery, &c. &c. By Geo. Mendenhall, M.D., Prof. Obstetrics, Medical College of Ohio, &c. Fifth Edition, revised and enlarged, 8vo. pp. 690. Philadelphia: Lindsay & Blakiston, 1857. (Received from the Publishers.)

Transactions of the Indiana State Medical Society at its Eighth Annual Session, held at Indianapolis, May 19th, 1857.

Exsection of the Entire Os Calcis. By J. M. Carnochan, M.D., Prof. Surgery New York Medical College, &c. (Received from the Author.)

Illustrated Scientific and Descriptive Catalogue of Achromatic Microscopes, manufactured by J. & W. Grunow & Co. New Haven, Conn. (Received from Prof. B. J. Silliman, Jr., M.D.)

A case of Fibrous Tumor of the Uterus, accompanied with Excessive Hemorrhage, successfully treated by Excision. By B. F. Barker, M.D., Prof. Midwifery New York Medical College. New York, 1857. (Received from the Author.)

Report of an Operation for Removing a Foreign Body from beneath the Heart. By E. S. Cooper, A.M., M.D. San Francisco, 1857. (Received from the Author.)

Human Histology in its Relations to Descriptive Anatomy, Physiology, and Pathology. With 434 illustrations. By E. R. Peaslee, A.M., M.D., Prof. Physiology and Pathology, N. York Med. College, &c. &c. 8vo., pp. 616. Philadelphia, Blanchard & Lea, 1857. (Received from the Publishers.)

Report on the Vital Statistics of the United States, made to the Mutual Life Insurance Company of New York. By James Wynne, M.D. New York: H. Bailliere. 1857.

### FOREIGN.

A Theoretical and Practical Treatise on Midwifery, including the Diseases of Pregnancy and Parturition, and the attentions required by the Child from Birth to the Period of Weaning. By P. Cazeaux, Adj. Prof. in Fac. Med. of Paris. Second American Edition, Translated from the Fifth French Edition, by W. R. Bullock, M.D. 8vo., with 140 illustrations. Philadelphia: Lindsay & Blakiston, 1857. (Received from the Publishers.)

Lectures on the Diseases of Women. By Chas. West, M.D., F.R.C.P., &c. Part I.—Diseases of the Uterus. 8vo. p. 316. Republished by Blanchard & Lea, Philadelphia, 1857. (Received from the Publishers.)

The Practice of Surgery. By James Miller, F.R.S.E., &c. Revised by the American Editor. Fourth American from the last Edinburgh Edition. 8vo. pp. 682. Philadelphia: Blanchard & Lea, 1857. (Received from the Publishers.)

Observations on the Human Crania contained in the Museum of the Army Medical Department, Fort Pitt, Chatham. By George Wilson, M.D. Dublin, 1857.

The Functions and Disorders of the Reproductive Organs in Youth, in Adult Age, and Advanced Life. By Wm. Acton. London, 1857.

God in Disease, or the Manifestations of Design in Morbid Phenomena. By J. F. Duncan. M.D. London, 1857. Pp. 323.

The Cattle Plague and Diseased Meat. Two Letters. By J. S. Gamgee. London, 1857. (Received from the Author.)

On the Function of the Thyroid Body. By O. Martyn, M.D., R.N. Proceedings of the Royal Society, Jan. 8, 1857.

Traité des Applications de l'Electricité a la Thérapeutique, Medicale et Chirurgicale. Par A. Becquerel, Médecin de l'Hopital de la Pitié. Paris.

### INDEX TO VOL. I.

Absorption by the large intestine, 447.
Abscess of the tibia, 113.
Abortion from patulous os uteri, 145.
criminal, case of, 142.
Acetate of lead in inverted nail, 119.
Address by R. La Roche, M.D., 883.
Africa, Central, ethnology of, 463.
Agnew's edition of London Dissector, notice of, 254.
Allen, Jas., M.R.C.S., on menstruation, 156.
Allen's Practical Anatomy, 254.
Almshouse Hospital, Blockley, 789.
American Med. Association, Transactions of, for 1856, 44.
American Med. Association, proceedings, for 1857, 617, 635.
American medical education, 301, 610.
literature, 379.
American surgeon, decorated by Russia, 798.
Ammonia and its preparations, 435.
Amussat's operation, example of, 409.
Amylene, 307, 775.
Anæsthesia, a new mode of, 307.
of uterus, 137.
Anatomy, Practical, by Wilson, notice of, 254.
by Allen, notice of, 254.
by Agnew, notice of, 254.
Physiological, by Todd and Bowman, notice of, 575.
study of legalized, 304.
Anæmia, by Ayres, 748.
Ancelon, M., on hooping-cough, 438.
Ancient Medicine, History of, by Watson, review of, 1.
by Gauthier, review of, 1.
Aneurisms and their Treatment, by Broca, review of, 211, 321.
Aneurism, popliteal, treated by compression, 281.
treatment of, by manipulation, 755.
Annan, Robert, on tetanus, 130.
Apnœa, treatment of, 436.
Arabian surgery, 311.
Arabia, diseases of, 705.
Armor, S. G., M.D., appointment of, 796.
Arteries, ligature of, by Gibbons, 78.
Arteria innominata, ligature of, 117.
Artificial Vichy water, 474.
Army med. staff, admissions into, 305.
Assafœtida in hooping-cough, 438.
Arsenical poisoning, 446.
Ascites and anasarca, 448.

Barclay, R. G., M.D., on diseases of Syria and Arabia, 705.
Bartlett on the Fevers of the United States, review of, 161.
Barton on Yellow Fever, notice of, 241.
Bamberger on pericarditis, 128.
Bayless, Dr. J. W., appointment of, 796.
Beck's Materia Medica, notice of, 60.
Belladonna applied to the breast, 462.
Bell, Dr. T. S., appointment of, 796.
Bemiss, S. M., M.D., on marriages of consanguinity, 97.
compliment to, 637.
Bernard on the temperature of the blood, 109.
Bernard's Experimental Physiology, review of, 529, 699.
Bertolet, P. G., M.D., on prolapsus uteri, 294.
Blackie, Geo. S., M.D., on Cretinism, notice of, 38.
appointment of, 797.
Blood, temperature and coagulation of, 109.
Blackman, Geo. C., M.D., notes to Velpeau's Surgery, 57.
on aneurism, 755.
on lagopthalmos, 426.
Blache, Dr., on chorea, 125.
Bladder, foreign body in, 413.
Blastema, Bowen on the, 719.
Blisters to cervix uteri, 783.
Blockley Hospital, disgraceful appointment to, 789.
Blaney, J. V. Z., M.D., appointment of, 476.
Blodgett on Climatology, notice of, 874.

Bozeman on urethro-vaginal and vesico-vaginal fistules, 576, 885.
Bozeman's operation in England, 157.
Bonpland, age of, 318.
Bowen, W. S., M.D., on the parent tissue, 719.
Boeck on syphilization, 758.
Boling, W. M., M.D., two surgical cases, 606.
Bowman's (Todd and) Physiology, notice of, 575.
Brown, J. B., on sterility, 455.
Broca on Aneurisms, review of, 211.
Breast, treatise on the, by Velpeau, review of, 193.
Bronzed skin and cirrhosed liver, 614.
Brickell, D. W., M.D., on menstruation, 453.
Butler, S. W., M.D., on lacerated perineum, 110.
Burning of the University of Louisville, 307.
Byford, W. H., M.D., on spina bifida, 120.

Cazeaux, P., treatise on Midwifery, &c., by, notice of, 883.
Calculus, intestinal, 127.
Campbell, Robt., M.D., on inverted toe-nail, 424.
H. J., M.D., appointment of, 638.
Cancer of the Breast, by Velpeau, review of, 193.
of the uterus, 454.
Treatment of, by Fell, 688.
Carriere's Climate of Italy, review of, 801.
Carswell, the late Sir Robert, notice of, 799.
Carbonic acid, a local anæsthetic, 137.
in the production of abortion, 139.
effect on the uterus, 139.
Catalogue of Pennsylvania Hospital Library, notice of, 880.
Cataract, lamellar, 743.
Chloroform in tetanus, 121.
Chorea, treatment of, 125.
Charcoal in cholera, 132.
Churchill on Diseases of Women, review of, 695.
Changes in medical schools, 796.
Chlorate of potash in ulcerations of mouth, 448.
in pertussis, 448.
Change of Life, by Tilt, notice of, 575.
Cirrhosis of liver, with bronzed skin, 604.
Cliff, D. B., M.D., on peritonitis, 299.
Climate, influence of, on tubercular disease, 801.
Climate of Pau, treatise on, by Taylor, review of, 801.
Italy, by Carriere, review of, 801.
Climatology of United States, treatise on, by Blodgett, notice of, 874.
Clinical Lectures on the Urinary Organs, by Todd, review of, 858.
Controversy about the late Mr. Liston, 154.
Comegy's translation of Renouard's History of Medicine, review of, 1.
Copaiba as an injection, 114.
Colon, stricture of, 409.
Consumption, night-sweats of, 433.
Coindet, Dr., injustice to, 311.
Colles' surgical cases, 763.
Concretions of prostate, 769.
Consumption treated with glycerine, 770.
Cock, Thos. F., on uterine hemorrhages, 778.
Comparative Physiology, by Jones, notice of, 697.
Colica pictonum, 605.
Coolidge, R.H., M.D., Report on the Sickness of the U. S. Army, review of, 561.
Craig on electric influence, 439.
Crania Britannica, review of, 665.
Crania, Catalogue of, by Meigs, notice of, 570.
Cretins and Cretinism, thesis on, by Blackie, notice of, 38.
Curiosities of mineral springs, 787.

Darley, B. G., M.D., on the taxis, 429.
Davis's Crania Britannica, review of, 665.
Dark side of war, 474.
Demarquay on vaginitis, 118.
Digestion, experiments on, by F. G. Smith, M.D., 598.
Diseases of the Female Sexual Organs, by F. Scanzoni, review of, 832.
Diseases of the Eye, by Dixon, review of, 351.
Urinary Organs, by R. B. Todd, review of, 858.
Ear, by Nottingham, notice of, 878.
Skin, by E. Wilson, notice of, 879.
Breast, by Velpeau, review of, 193.
Dickson, S. N., M.D., Elements of Medicine, review of, 161.
Dislocation of the thumb, new mode of reduction, 113.
tibia, 298.
femur into sacro-sciatic notch, reduced by manipulation, 933.
Doe, J. N., M.D., on dislocation of the thumb, 113.
Dog-latin, 158.
Duncan, R. V., M.D., on puerperal fever, 931.
Dupierris, M., M.D., Report on the Medical Topography of Havana, review of, 870.
Dupierris, M., M.D., on the treatment of uterine hemorrhages, 83.
Dupuytren, reminiscences of, 312.
Dysentery treated with iodine, 451.
Dyspepsia, lactic acid in, 772.

Ear, Diseases of, by Nottingham, notice of, 878.

Editorial labor, 148.
Editing medical books, 639.
Education, medical, American system of, 301, 610.
Effects of Climate on Tuberculous Disease, by Lee, review of, 801.
Elements of Operative Surgery, by Velpeau, notice of, 57.
Elements of Medicine, by Dickson, review of, 161.
Electricity a cause of disease, by Littell, 390.
by Craig, 439.
Ellis, Mr., on abortion, 145.
Encephaloid, tumor of, in cranium, 929.
Erysipelas and puerperal fever, 600.
Esculapius the son of Apollo, 475.
Ethnology of Central Africa, 403.
Eulogium on Roux, 312.
Excision of os calcis, 762.
Eye, Diseases of, by Dixon, review of, 501.
Eyre, Sir Jas., obituary of, 799.

Fell, on the Treatment of cancer, 688.
Female Sexual Organs, treatise on, by Scanzoni, review of, 832.
Female med. colleges and doctors, 942.
Femur, dislocation of six months' standing reduced, 290.
Femur, dislocation of, into sciatic notch, reduced by manipulation, 933.
Fenner, C. S., M.D., on granular conjunctivitis, 66
Fevers of the United States, treatise on, by Bartlett, review of, 161.
Fever, Yellow, at New Orleans, treatise on, by Barton, review of, 241.
Finnell, J. C., M.D., on criminal abortion, 142.
Fischer's Catalogue of Pennsylvania Hospital Library, 880.
Fistules, urethro-vaginal and vesico-vaginal, by Bozeman, 576, 885.
Foreign text-books, 797.
Fricke, C. J., M.D., on bronzed skin with cirrhosed liver, 604.

Gadbury, W. Y., M.D., election of, 476.
Gangrene, senile, by Markoe, 431.
German Universities, students at, 798.
Gibbons, T.P., M.D., on ligation of arteries, 78.
Gibbs, O. C., M.D., on the economical use of quinia, 79.
Gibbs, O. C., M.D., on pregnancy, after long-suppressed menstruation, 741.
Glycerine and tannin in vaginitis, 118.
in consumption, 770.
Gliddon, Geo. R., on Ethnology, review of, 665.
Gosselin on ranula, 114.
Gonorrhœa treated by injections of copaiba, 114.
Granular conjunctiva, by Fenner, 66.
Graham, B. F., M.D., on quinoidine, 412.
Graduates, medical, for 1857, 476.
Graham, Thomas, M.D., on strabismus (plagiarized from Critchett), 259.
Gross, S.D., M.D., a new tourniquet, by, 96.
on dislocation of tibia forward, 298.
Report on American Medical Literature, review of, 319.
Pathological Anatomy, treatise on, notice of, 702.
case of lead pencil in the bladder, by, 413.
Gross, S. W., M.D., case of scirrhus of liver, 414.
Guerin on Nervous Irritation, review of, 641.
on Intermittent Fevers, review of, 641.
Guy's Hospital Reports for 1856, review of, 548.
Gymnastic exercise in chorea, 125.

Hall, Dr. Marshall, on resuscitation of stillborn infants, 146.
Hall, Dr. Marshall, notice of death of, 952.
Hamilton, F., M.D., on abscess of tibia, 113.
Hammond, Wm. A., M.D., on excretion of phosphoric acid by the kidneys, 730.
Hardin, J. M., M.D., on transverse presentations of fœtus, 73.
Hare-lip, new suture for, 309.
Havana, Medical Topography of, 870.
Hemorrhage, uterine, iodine in, 83.
secondary, by Cock, 778.
ice in, 451.
cancer of cervix, 454.
from socket of tooth, 606.
Hentz, Chas. A., M.D., on hooping-cough, 285.
Henry, G. R., M.D., on stricture of colon, 409.
on dislocation of femur, 933.
Headland, Dr. F. W., on coagulation of blood, 109.
Hernia, taxis in, 429.
inguinal, 766.
strangulated, with inflamed testis, operation in, 767.
on the back, 768.
ventral, rare case of, 768.
History of Medicine, treatise on, by Renouard, review of, 1.
History of medicine, treatise on, by Watson, review of, 1.
History of medicine, by Gauthier, review of, 1.
Hinkle, F., M.D., on traumatic tetanus, 121.
Hinds, Dr., on arsenical poisoning, 446.
Hildreth, E. A., M.D., on introduction of ice into uterus, 451.

Historical Sketch of Quarantine, by W. Jewell, M.D., review of, 356.
Hollingsworth, Dr. S. L., and the Medical Examiner, 152.
Homœopathists in Massachusetts Medical Society, 155.
Horseflesh used as food, 159.
Hooping-cough, by Hentz, 285.
treated with assafœtida, 438.
Holland, Sir H., Medical Notes, review of, 368.
Howell, John D., M.D., notice of death of, 799.
Hogg, Jabez, treatise on the Microscope by, review of, 571.
Huston, Prof., resignation of, 638.
Hunt, W., M.D., notes to Wilson's Dissector, by, notice of, 254.
Hullihen, S. P., M.D., notice of death of, 478.
Hydrocele, injection of, 766.
resulting from hernia, 766.
mistaken for hernia, 767.

Ice, introduction of, into uterus, 451.
Iceland moss gelatinized with cod-liver oil, 450.
Indiana State Medical Society's Transactions, notice of, 61.
Intestinal calculus, very large, 127.
Inversio uteri, cases of, by Bertolet, 294.
Inverted toe-nail, treatment of, 424.
Intestine, large, absorption from, 447.
Intermittence in Health and Disease, treatise on, by Pallas, review of, 641.
Intermittent Fevers, treatise on, by Guerin, review of, 641.
Indigenous Races, treatise on, by Nott and Gliddon, review of, 665.
Iodine in uterine hemorrhages, 83.
in dysentery, 451.
in vomiting in pregnancy, 451.
in ovarian dropsy, 429.
in large doses, mode of administering, 450.
Iron, perchloride of, as a hemostatic, 45.
in panniform keratitis, 117.
Isambert on chl. potass in pertussis, 448.

Jefferson Medical College, change in, 795.
Jewell, W., M.D., on the mortality of Philadelphia, 268, 415, 934.
Jewell, W., M.D., Address on Quarantine, review of, 356.
Journalism, medical, in Paris, 475.
Jones, Joseph, M.D., Physiological Investigations, notice of, 697.
Johns, Robert, on blisters to cervix uteri, 783.

Kane, E. K., M.D., notice of death of, 478.
Kempf, M., M.D , on ovariotomy, 738.
Kirkes, W. S., Manual of Physiology, notice of, 704.
Kidneys, excretion of phosphoric acid by, 730.
Kluge, J. P., M.D., encephaloid tumor in cranium, 929.
Kneeland's surgical statistics of Massachusett's General Hospital, 115.
Kunkler, G. A., M.D., on colica pictonum, 605.
on prolapsus uteri, 927.

Laceration of perineum, 110, 288.
Lactic acid in dyspepsia, 772.
La Roche, R., M.D., Address by, notice of, 883.
Lagophthalmos, case of, 426.
Lamellar cataract, case of, 743.
Lead pencil in the bladder, 413.
Lee, Ed., Essay on Climate in Tubercular Disease, review of, 801.
Lehman on stricture of the uterus, 140.
Lessons in Experimental Physiology, by Bernard, review of, 529.
Levergood, J., M.D., on puerperal fever, 600.
Ligation of arteries, 78.
Ligation of innominate artery, successful, 117.
Libel, suit for, by Dr. Goadby, 157.
Library Pennsylvania Hospital, Catalogue of, notice of, 880.
Littell, A., M.D., on effects of electricity in the production of disease, 390.
Livingston, Dr., 309.
Liver, scirrhus of, in an infant, 414.
Louisville University, burning of, 306.

Martin, J. R., M.D., Treatise on Tropical Climates, review of, 481.
Marriages of consanguinity, by Bemiss, 97, 797.
Maissonneuve on hemostatics, 115.
Manipulation, reduction of dislocation by, 290, 933.
treatment of aneurism by, 755.
Markoe, Dr., on senile gangrene, 431.
Manual of Physiology, by Kirkes, notice of, 704.
Maund, John, M.D., case of poisoning by turpentine, 772.
Materia Medica, treatise on, by Wood, notice of, 65.
Materia Medica, treatise on, by Beck, notice of, 60.
Materia Medica, treatise on, by Mitchell, notice of, 884.
Massachusetts General Hospital, surgical statistics of, 115.
Mayo, Dr., election of, 308.
McClintock, Dr. James, election of, to Blockley Hospital, 787.
Medical Notes and Reflections, by Holland, review of, 368.
Medical Literature, American, by Gross, review of, 379.
Medicine, History of, by Renouard, review of, 1.
by Watson, review of, 1.

Medicine, History of, by Gauthier, review of, 1.
Spanish, 467.
in Syria and Arabia, 705.
Principles of, by Williams, review of, 877.
Medical Topography of Havana, report on, review of, 870.
teachings and opportunities in Philadelphia, 948.
Meeting of American Medical Association at Nashville, 794.
Menstruation, decline of, 142.
physiology of, 453.
Meigs, Chas. D., M.D., treatise on Obstetrics, notice of, 258.
Meigs, J. A., M.D., Catalogue of Crania, notice of, 570.
appointment of, 305.
Metcalfe, Saml., M.D., notice of death of, 477.
Microscope in diagnosis of phthisis, 123.
treatise on, by Hogg, notice of, 571.
Midwifery, &c., treatise on, by Cazeaux, notice of, 883.
Miller, Hugh, notice of death of, 319.
Milk, secretion of, arrested by belladonna, 462.
Miller, James, treatise on the Practice of Surgery, notice of, 882.
Mineral springs, virtues of, 631.
curiosities of, 787.
of the Pyrenees, treatise on, review of, 801.
Mitchell, T. D., election of, 796.
treatise on Materia Medica, notice of, 884.
Montgomery, W. F., M.D., treatise on the Signs and Symptoms of Pregnancy, review of, 249, 694.
Monomania, suicidal, 472.
Morroh, C., M.D., on exsection of os calcis, 762.
Morals of the Viennese, 950.
Mortality, statistics of Philadelphia, for 1856, 268.
Mortality, statistics of Philadelphia, for 1857, 415, 934.

Nail in the flesh, 119.
Nancrede, J. G., M.D., notice of death of, 478.
Newton, Prof. S. M., M.D., resignation of, 638.
Night-sweats, treatment of, 433.
Nottingham, John, treatise on the Ear, notice of, 878.
Nott, Dr. J. C. (and Gliddon) treatise on the Indigenous Races, review of, 665.
Nott, Dr. J. C., appointment of, 476.

Obstetrics, the Science and Art, by Meigs, notice of, 258.
Obliteration of the portal vein, 108.
Œdema of the prepuce, 765.
Oil of turpentine, poisoning by, 772.
Operative Surgery, Elements of, notice of, 57.
Opium trade, 466.
Ophthalmological congress, 797.
Oré, M., on obliteration of portal vein, 103.
Ovarian dropsy, treatment of, 429.
discussion upon, 456.
tumor, excision of, 157, 738.

Paris, Dr. John, notice of death of, 320.
Paralysis of orbicularis palpebrarum, 426.
Parent tissue and blastema, 719.
Paraphimosis, treatment of, 764.
Patulous os uteri a cause of abortion, 145.
Pathological Anatomy, Elements of, by Gross, notice of, 702.
Pathological Society of Philadelphia, organization of, 950.
Peixoto, M., successful ligature of innominate artery, by, 117.
Pendleton, E. M., M.D., on spina bifida, 428.
Penis, tumor of, 765.
Pennsylvania Hospital Library Catalogue, notice of, 880.
Medical Society, proceedings, 628, 633.
Perineum, laceration of, 110, 288.
Perineal incision, by Gross, 413.
Perchloride of iron as a hemostatic, 115.
in panniform keratitis, 117.
Pericarditis, by Bamberger, 128.
the result of criminal abortion, 142.
developed by pregnancy, 299.
Periodicity, a review, 641.
Phthisis, diagnosis of, by the microscope, 123.
Philadelphia County Medical Society, officers for 1857, 306.
Hospital, Blockley, 789.
Physiology, Experimental, by Bernard, review of, 529, 699.
Manual of, by Kirkes, notice of, 704.
Physiological Anatomy, by Todd and Bowman, notice of, 575.
Physician's Pocket-book, &c., by Wythes, notice of, 569.
Visiting List, notice of, 884.
Phosphoric acid, excretion of, by kidneys, 730.
Pitting in small-pox, prevention of, 434.
Plagiarism, 637, 948.
Pneumonia, treatment of, 602.
Popliteal aneurism, treated by compression, 281.
artery, perforation of, by exostosis, 608.
Poisoning by strychnine, 434.
by colored wall-paper, 446.
by turpentine, 772.
Pope, C. J., M.D., on pneumonia, 602.
Potash, chlorate of, in membranous stomatitis, 448.
Practice of Surgery, treatise on, by Smith, review of, 26.

Practice of Surgery, treatise on, by Miller, notice of, 882.
Presentations, transverse, of fœtus, 73.
Premature labor induced by carbonic acid, 139.
Pregnancy, Signs and Symptoms, by Montgomery, review of, 249, 694.
Diseases of, by Churchill, notice of, 695.
in suppressed menstruation, 741.
Prepuce, œdema of, 765.
Principles of Medicine, treatise on, by Williams, notice of, 877.
Prostate, concretions of, 769.
Prolapsus uteri, 294, 927.
Psychology of acute rheumatism, 922.
Puerperal fever and erysipelas, 600.
treated with iron and opium, 931.

Quackery, swindling, 153.
Quarantine, History of, by Jewell, review of, 356.
convention, proceedings, 623, 636.
Quinoidine, a substitute for quinine, 412.
Quinia, economical use of, 79.
Quis custodet custodes, 301.

Races, Indigenous, by Nott and Gliddon, review of, 665.
Ranula, treatment of, 114.
Ready method for resuscitating the asphyxiated, 436.
Reduction of femoral dislocation by manipulation, 290, 933.
of dislocated thumb by a new method, 113.
Removal of tumors by a new method, 429.
Reminiscences of Dupuytren, 312.
Renouard's History of Medicine, review of, 1.
Rheumatism, acute, psychology of, 922.
Robert, M., and others, on amylene, 775.
Rochard, M. Jules, on Sea-Voyages in Consumption, review of, 801.
Roux, eulogium on, 312.
Rules for resuscitating stillborn infants, 146.
Rupture of the urethra, 764.
Rupture of the perineum, 110, 288.

Scanzoni, Dr. F. W., Diseases of the Female Sexual Organs, review of, 832.
on carbonic acid to produce abortion, 139.
Scirrhus of the liver in an infant, 414.
Secretion of bile, experiments on, 108.
Senile gangrene, 431.
Sickness and Mortality of U. S. Army, report on, review of, 561.
Signs and Symptoms of Pregnancy, by Montgomery, review of, 249, 694.
Simpson, Prof., on carbonic acid as a local anæsthetic, 137.
on a new mode of removing tumors, 429.
Skin, Diseases of, by Wilson, review of, 879.
Smith, H. H., M.D., treatise on Surgery by, review of, 26.
Smith, Francis G., M.D., experiments on digestion, 598.
Spanish medicine, 467.
Speculum, use of, 783.
Sphere of editorial labor, 148.
Spina bifida, three cases of, 428.
Strabismus, a new mode of operating for (a plagiarism), 259.
Statistics of Massachusetts General Hospital, 115.
Stillborn infants, resuscitation of, 146.
Sterility induced by diseases of rectum, 455.
Stricture of the uterus, 140.
of the colon, 409.
Strychnine, treatment of poisoning by, 434.
its uses and abuses, 444.
Students at the German Universities, 798.
Surgery, treatise on, by Smith, review of, 26.
by Velpeau, notice of, 57.
Practice of, by Miller, notice of, 882.
Suicidal monomania, 472.
Superannuated medical teachers, 310.
Syphilization, by Boeck, 758.

Taggart, W. H., M.D., appointment of, 306.
Tannin and glycerine in vaginitis, 118.
Taxis, new mode of employing, 429.
Taylor, M. A., M.D., treatise on the Climate of Pau, review of, 801.
Testis, misplaced, case of, 765.
Tetanus, traumatic and idiopathic, 121, 130.
Text-books, 797.
Temperature of blood, 109.
Therapeutics and Pharmacology, by Wood, notice of, 65.
Thumb, dislocation of, 113.
Thurman, John, M.D., Crania Britannica, review of, 665.
Tibia, dislocation of, forward, 298.
chronic abscess of, 113.
Tilt, E. J., M.D., on the Change of Life, notice of, 575.
Toe-nail, inversion of, 424.
Todd, Robert B., M.D., on the Urinary Organs, review of, 858.
Todd and Bowman's Physiological Anatomy, notice of, 575.
Topography of Havana, 870.
Tourniquet, a new form of, by Gross, 96.
Trabue, B. F., M.D., case of lacerated perineum, by, 288.
Transactions of American Medical Association for 1856, notice of, 44.
Transactions of Indiana State Medical Society, notice of, 61.
Transverse presentations of fœtus, 73.
Tubercular Diseases, Effect of Climate on, 801.

Tumors, removal of, by a new method, 429.
Turpentine, poisoning by, 772.

Uterine hemorrhage, a new mode of treatment of, 83.
ice in, 451.
secondary, 778.
diseases, local anæsthetics in, 137.
Uterus, gravid, effects of carbonic acid on, 139.
stricture of, 140.
inversion of, 294.
cancer of, 454.
blisters to neck of, 783.
Ulcerations of mouth and throat, chlorate potass in, 448.
Urinary Organs, Diseases of, by Todd, 858.
Urethro-vaginal and vesico-vaginal fistules, 576, 885.

Vesico-vaginal fistules, by Bozeman, 576, 885.
Von Mauthner, visit of, to Philadelphia, 950.
Voss, Dr., visit of, to Philadelphia, 950.

Ward, Dr. O. G., on anemia, 435.
Weber, G. C. E., M.D., appointment of, 306.
Williams, C. J. B., treatise on Principles of Medicine, notice of, 877.
Wilson, E., on diseases of the skin, notice of, 879.
Wheelock, Albert T., M.D., on the psychólgy of acute rheumatism, 922.
Wood's Therapeutics and Pharmacology, notice of, 65.
Wood, Mr. A. G., on a new hare-lip suture, 309.

Yellow Fever, Cause and Prevention of, by Barton, notice of, 241.

